# Prophylactic Closed Suction Drainage Is Irrelevant to Accelerated Rehabilitation after Open Reduction and Internal Fixation for Closed Distal Femur Fractures

**DOI:** 10.1111/os.12812

**Published:** 2020-10-12

**Authors:** Jun‐zhe Zhang, Kuo Zhao, Jun‐yong Li, Hong‐yu Meng, Yan‐bin Zhu, Ying‐ze Zhang

**Affiliations:** ^1^ The Third Hospital of Hebei Medical University Shijiazhuang China; ^2^ Key Laboratory of Biomechanics of Hebei Province Orthopaedic Research Institution of Hebei Province Shijiazhuang China; ^3^ Department of Orthopaedic Surgery The Second Hospital of Shijiazhuang City Shijiazhuang China

**Keywords:** Distal femoral fractures, Drainage, Open reduction and internal fixation, Randomized controlled trial

## Abstract

**Objective:**

To investigate whether closed suction drainage (CSD) is related to accelerated rehabilitation of patients after open reduction and internal fixation (ORIF) for closed distal femur fractures.

**Methods:**

This study was a prospective, randomized controlled clinical trial. Between October 2018 and June 2020, 160 closed distal femur fracture patients who were prepared for ORIF were prospectively randomized into two groups: a CSD group with the mean age of 57.91 ± 14.38 years (32 [40%] men and 48 [60%] women) and a non‐CSD group with the mean age of 59.73 ± 17.55 years (27 [34%] men and 54 [66%] women). Wound visual analogue scale (VAS) pain scores, peri‐wound skin temperature, hematocrit (Hct), hemoglobin (Hb) concentrations, hidden blood loss (HBL), dressing change, period of wound oozing, postoperative blood transfusion, and length of postoperative hospital stay were recorded. Postoperative wound complications, namely wound infections, wound haematoma, wound dehiscence, erythema of wound, and lower limb deep vein thrombosis (DVT) were collected. All the patients were administrated by a single surgical team and followed up for 1 month after the ORIF.

**Results:**

The patients without CSD were identified with lower peri‐wound skin temperature and wound VAS pain scores during the first three postoperative days (36.69 ± 0.33 *vs* 36.86 ± 0.38 °C, *P* = 0.002; 1.88 ± 0.82 *vs* 3.15 ± 1.15, *P* = 0.000). However, both the peri‐wound skin temperature and wound VAS pain scores did not differ significantly between the two groups on the fifth postoperative day. In addition, patients with CSD had a longer length of postoperative hospitalization time (11.45 ± 5.95 *vs* 9.78 ± 4.64 days, *P* = 0.049). There was no statistically significant difference between CSD and non‐CSD groups within 1 month after the ORIF regarding blood loss, period of wound oozing, and postoperative complications, such as incidence of wound infection, haematoma, erythema, dehiscence, and lower limb DVT.

**Conclusion:**

Prophylactic CSD after primary ORIF for closed distal femur fractures not only had no significant advantage to minimize blood loss and wound complications, but increased local inflammation and postoperative hospital stay, and thus we suggest that prophylactic CSD after primary ORIF for closed distal femur fractures is not recommended for optimized clinical pathways and accelerated recovery.

## Graphical abstract

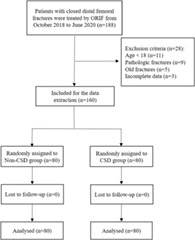

Non‐use of prophylactic drains after primary surgery for closed distal femur fractures was related to less local inflammation and shorter postoperative hospital stay. Prophylactic drains had no significant advantage to minimize blood loss and wound complications, and we suggest that prophylactic drains after surgery are not recommended for this type fractures.

## Introduction

Closed suction drainage (CSD) has been routinely applied in orthopaedic and trauma surgery. The CSD was used for the purpose of a relatively lower infection risk and less potential dead space of orthopaedic wounds, which was first proposed by Waugh in 1961^1^. Few would doubt the therapeutic effects of the CSD to inhibit abscess, fistulas, or necrotic debris, but there still exists a lack of evidence‐based guidelines to confirm the advantages of “prophylactic” CSD for the potential cause that it reduces wound hematomas, which can increase wound tension and the incidence of infection in most surgical sites. According to previous research reports, the CSD may also be associated with control of systemic symptoms (fever, anemia, etc.) and local symptoms (redness, pain, non‐healing wounds, etc.)^2^, ^3^, ^4^, ^5^, ^6^. In addition, some documents have demonstrated that CSD contributes to decreasing the rate of wound complications such as wound erythema, ecchymosis, and dehiscence^7^, ^8^.

However, an adverse effect of prophylactic CSD is that they may become a potential infection source and act as an infection path into the depths of the wound^9^. Some literature indicates that CSD plays an important role in the development of surgical site infection in orthopaedic trauma patients^10^, ^11^. Besides this, according to a meta‐analysis of the efficacy of CSD in orthopaedic patients, postoperative blood transfusion was required more frequently in those patients with drainage used^12^. No statistically significant difference regarding the incidence of wound infection, haematoma, dehiscence, or re‐operations was found between the groups with and without CSD. In consideration of few beneficial local effects of CSD, additional cost can also be one reason for the reduction of prophylactic CSD in one‐stage orthopaedic surgery. Furthermore, on rare occasions, CSD may be displaced or removed prematurely by confused patients pulling on them. As both perioperative fluid management and hemostatic techniques advance, intraoperative bleeding and transfusion requirement have been markedly decreased^13^, ^14^. Therefore, the effect of CSD on enhancing recovery after surgery and length of hospital stay remains controversial.

Distal femur fractures are relatively rare but severe in orthopaedic trauma, comprising approximately 8.65% of all femoral fractures and 0.8% of total body fractures in Chinese adults^15^. In young and middle‐aged adults, the majority of those fractures result from high‐energy injuries and easily involve the popliteal artery and articular surface. Thus, the distal femur fractures are often accompanied by greater blood loss and local hematomas. To prevent the formation of wound haematomas and enhance recovery after surgery, CSD has been routinely used after open reduction and internal fixation (ORIF) for distal femur fractures in our centre. However, we had the impression that CSD was correlated to increased complaints about foreign body sensation, dressing change, and wound pain. At present, most studies have focused on CSD in spinal surgery and arthroplasty for hip, knee, or shoulder^2^, ^3^, ^4^, ^5^. Little attention has been paid to the CSD in early wound recovery after one‐stage ORIF for closed distal femur fractures, especially in the past 5 years. No standardized protocol was established for the use of CSD after ORIF for closed distal femur fractures. In addition, the introduction of rapid tracking of clinical pathways and early rehabilitation after surgery also made prophylactic CSD as an issue worth discussing. Furthermore, it is significant to quantitatively evaluate the local effects of CSD during postoperative hospital stay to address the issues that previous studies have not clearly resolved.

Therefore, we performed a prospective clinical randomized controlled trial to more specifically and quantitatively assess the systemic and local efficacy of prophylactic CSD in early recovery of surgical site after one‐stage ORIF for closed distal femur fractures. We hypothesized that: (i) the prophylactic CSD would be correlated to more postoperative wound pain and higher peri‐wound skin temperature; (ii) the prophylactic CSD would have no significant advantage to minimize blood loss and wound complications within 1 month after the ORIF; and (iii) non‐use of prophylactic CSD would contribute to shorter hospitalization time.

## Methods

### *Study Design*

From October 2018 to June 2020, a prospective clinical randomized controlled trial including consecutive patients undergoing one‐stage ORIF for acute closed distal femur fractures was conducted at the Third Hospital of Hebei Medical University. The study protocol was conducted according to the Declaration of Helsinki and was approved by the Institutional Review Board (NO 2018–026‐1), and all the participants provided written informed consent. The exclusion criteria were listed as follows: (i) age less than 18 years; (ii) old fractures (>21 days from initial injury); (iii) open or pathological fractures; (iv) simultaneous bilateral ORIFs or revision surgery; (v) history of femur surgery and deep vein thrombosis (DVT).

**Fig. 1:**
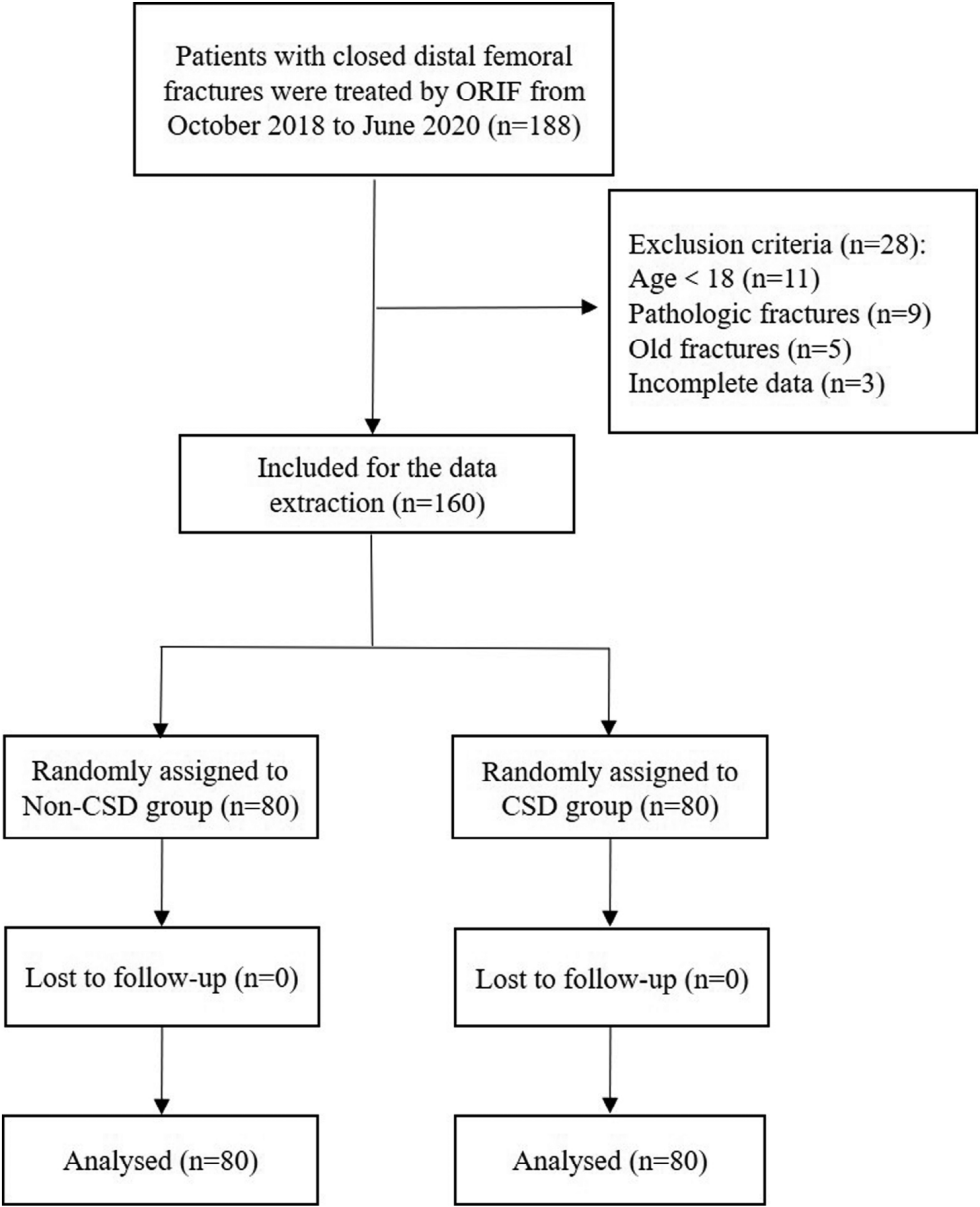
The flow chart for the selection of study participants.

As presented in Fig. 1, a total of 160 participants with a diagnosis of closed distal femoral fracture were evenly allocated to two groups: a CSD group and non‐CSD group. All the data were collected by four well‐trained investigators. Investigators followed the patients closely by morning work rounds and reviewed patients' clinical data. The suture site was observed by researchers starting from the first day after ORIF until hospital discharge. All the patients were followed for wound complications about 1 month postoperatively without lost to follow‐up. Patients with suspected wound complications were requested to return for definitive diagnosis and treatment.

### *Surgical Criteria*

**Fig. 2:**
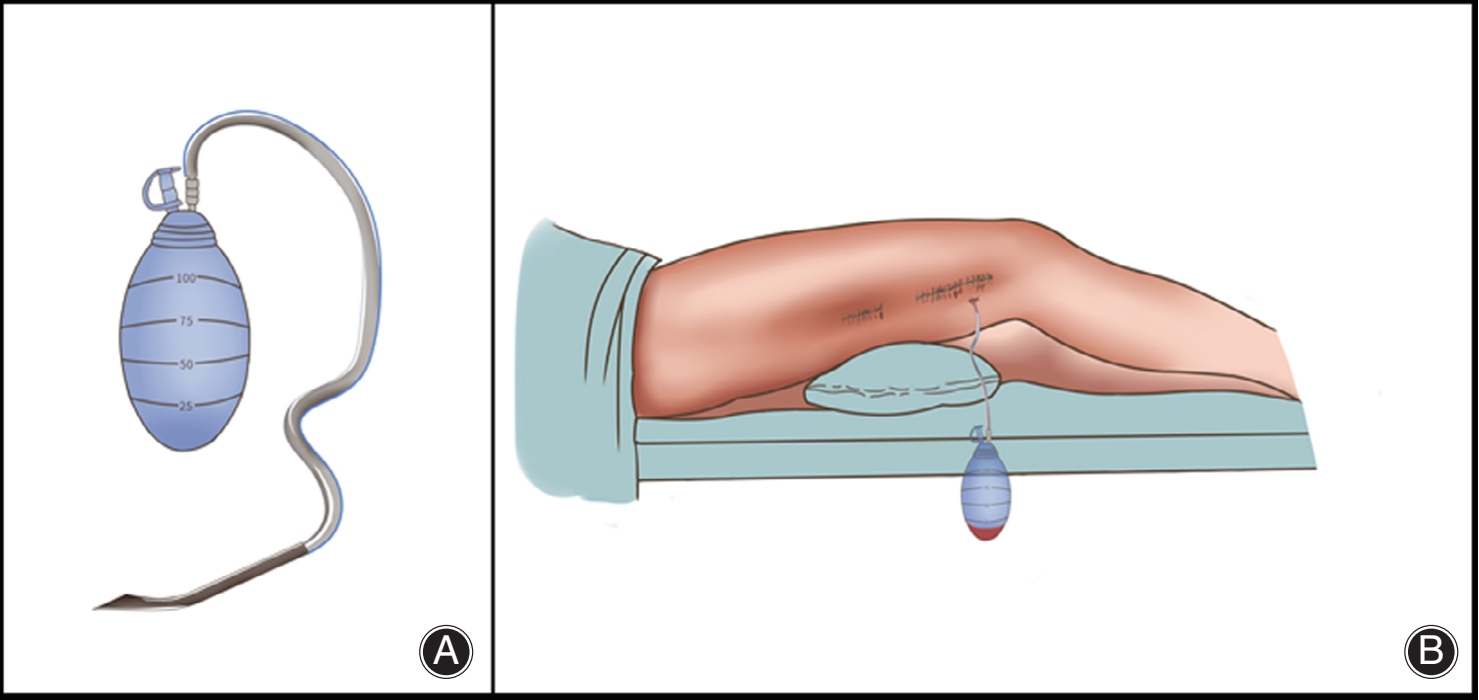
Two representative diagrams of the patient with a closed suction drainage (CSD). (A) It is a unified CSD with specificity: SY‐Fr16‐C, 100 mL. (B) The CSD was placed under the deep fascia before the incision was sutured closed.

All the ORIFs were carried out under general anesthesia in a supine position by a single surgical team in the same operating room. A tourniquet was inflated to 260 mm Hg before skin incision until skin closure. An anterolateral or anteromedial distal femoral approach for surgical exposures were uniformly chosen in all cases. The major component used in the ORIFs was the same kind of locking compression plates. As is shown in Fig. 2, the CSD (Specificity: SY‐Fr16‐C, 100 mL; Bainus Medical, Shandong, China) was placed under the deep fascia. After irrigation, the incision was sutured closed following a compression dressing.

### *Postoperative Management*

The CSD maintained patency and was removed 48 h after operations. Antibiotic prophylaxis (2.0 g cefazolin) was injected intravenously 30 min prior to surgery. All cases were managed and early rehabilitation exercises of quadriceps muscle were performed. Blood transfusions were performed according to the criteria of hemoglobin (Hb) values <70 g/L or 70–100 g/L with symptoms of severe anemia. To prevent DVT, low‐molecular‐weight heparin (LMWH) was administered within 24 h of presentation. The LMWH administered was enoxaparin (40 mg daily). If wound dressing was soaked with exudate, the dressing was directly changed.

### *Outcome Measurements*

During the study period, detailed variables of interest were collected and divided into four aspects.

### *Demographic and Fracture‐related Variables*

Demographic variables included age (years), gender, height (m), weight (kg), and body mass index (BMI, kg/m^2^). The BMI is equal to the weight divided by the square of height. Fracture‐related variables included affected side and relevant fracture type according to Arbeitsgemeinschaft für Osteosynthesefragen/Orthopaedic Trauma Association (AO/OTA) classification system. Differences between the two groups were not statistically significant with respect to demographic characteristics, involving age, gender, BMI, side, and relevant fracture type according to AO/OTA classification system, which are shown in Table 1.

**TABLE 1:** Summary of demographic data

Variables	Patient with CSD (*n* = 80)	Patient without CSD (*n* = 80)	*P* value
Age (mean±SD, years)	57.91 ± 14.38	59.73 ± 17.55	0.476*
BMI (mean±SD, kg/m^2^)	26.39 ± 4.65	25.97 ± 3.90	0.939^†^
Gender(male/female)	32/48	27/53	0.413^‡^
Side (left/right)	48/32	46/34	0.748^‡^
Fracture type			0.585^‡^
AO type A	44	49	
AO type B	9	10	
AO type C	27	21	

AO, Arbeitsgemeinschaftf‐ür Osteosynthesefragen; BMI, body mass index; CSD, closed suction drainage.

* Student *t* test.

† Mann–Whitney *U* test.

‡ Pearson Chi‐Square test.

### *Operation‐related and Clinical Outcomes*

Operation‐related variables included preoperative stay, postoperative stay, intraoperative blood loss (mL), operation duration (minutes), and the American Society of Anesthesiologists (ASA, I‐V) classification system^16^. Wound pains were quantified by a visual analog scale (VAS, 0 cm, no pain; 10 cm, worst possible pain imagined) immediately before ORIF and within seven consecutive days after surgery. Peri‐wound skin temperatures were measured with an infrared thermometer at four uniform distribution sites around the wound. The temperatures were recorded at 12:00 a.m. for seven consecutive days after ORIF.

### *Perioperative Blood Loss*

The values of hemoglobin (Hb) and hematocrit (Hct) were recorded from the day before operation to the fifth postoperative day (POD 5). With demographic factors such as gender, height, weight, and transfusion amount taken into calculation, the gross equation quantifies actual postoperative blood loss^17^. Estimated total blood loss was obtained using the gross formula according to the decrease of Hct the day before operation to postoperative day 3. The hidden blood loss (HBL) was finally calculated by subtracting the drained blood loss from the estimated total blood loss. Patients requiring transfusion after ORIF were also recorded.

### *Complications*

Postoperative wound complications including wound infections, wound haematoma, wound dehiscence, erythema of wound, and DVT were recorded. The Doppler ultrasonography was performed before and after surgery for DVT confirmation.

## Statistics

Statistical analysis was performed by SPSS version 25.0 (IBM Corp., Armonk, NY, USA). The continuous variables are shown as mean ± standard deviation (SD). The distributions of all data were evaluated for normality by the Shapiro–Wilk test. A Whitney *U* test or *t* test was used to compare continuous variables between CSD and non‐CSD groups depending on the equal variance and normality distribution status. Pearson's chi‐square or Fisher's exact test was utilized to analyze categorical variables. Normally, a *P* < 0.05 was considered statistically significant. Sample size was estimated from the length of postoperative hospital stay. A minimum difference of 1 day according to postoperative hospital stay was considered to be of clinical significance. A sample size was 64 in each group with a power of 80%, which was analyzed by G*Power 3.1.9.2.

## Results

### *Preoperative and Intraoperative Outcomes*

For the 160 patients (80 in each group), the average age was 58.82 ± 16.01 years (59 [37%] men and 101 [63%] women), and the average BMI was 26.18 ± 4.29 kg/m^2^. No statistical differences of the two groups were identified with respect to preoperative Hb, Hct, VAS pain score, peri‐wound skin temperature, ASA, intraoperative blood loss, and surgical duration (Table 2).

**TABLE 2:** Preoperative and intraoperative characteristics in patients with and without CSD

Variables	Patient with CSD (mean ± SD) (*n* = 80)	Patient without CSD (mean ± SD) (*n* = 80)	*P* value
Preoperative Hb (mean±SD, g/L)	116.09 ± 13.90	118.97 ± 13.42	0.209^†^
Preoperative Hct (mean±SD, %)	34.61 ± 4.12	35.10 ± 3.78	0.507^†^
Preoperative VAS pain score (mean±SD)	5.86 ± 1.19	5.68 ± 0.81	0.372^†^
Preoperative Peri‐wound skin temperatures (mean±SD, °C)	36.67 ± 0.37	36.59 ± 0.33	0.144*
ASA			0.324^‡^
I‐II	48	54	
III‐V	32	26	
Preoperative stay (mean±SD, days)	6.65 ± 4.02	7.21 ± 4.73	0.578^†^
Intraoperative blood loss (mean±SD, mL)	465.63 ± 295.55	428.13 ± 245.57	0.549^†^
Surgical duration (mean±SD, min)	158.56 ± 54.90	150.75 ± 64.69	0.131^†^

ASA, American Society of Anesthesiologists; CSD, closed suction drainage; Hb, hemoglobin; Hct, hematocrit; VAS, visual analog scale.

* Student *t* test.

^†^ Mann–Whitney *U* test.

^‡^ Pearson Chi‐Square test.

### *Postoperative Outcomes*

Postoperative variables were shown in Table 3. In the CSD group, leakage from the CSD hole persisted about 2.02 ± 0.32 days and average drainage volume was 172.06 ± 108.29 mL. There was no statistical significance found between the CSD and non‐CSD groups regarding dressing change. While the period of wound oozing tended to be longer in the non‐CSD group than the CSD group, the difference remained not statistically significant (*P* = 0.087). The average length of postoperative hospital stay was 9.78 ± 4.64 and 11.45 ± 5.95 days in the non‐CSD and CSD groups; the difference was statistically significant (*P* = 0.049).

**TABLE 3:** Details of postoperative variables according to study groups (mean±SD)

Variables	Patient with CSD (*n* = 80)	Patient without CSD (*n* = 80)	*P* value
Drainage (mL)	172.06 ± 108.29	N/A	
Period of drain hole leakage (days)	2.02 ± 0.32	N/A	
Dressing change (mL)	3.50 ± 1.60	3.61 ± 1.49	0.606^†^
Period of wound oozing (days)	3.23 ± 1.37	3.61 ± 1.48	0.099^†^
Postoperative hospital stays (days)	11.45 ± 5.95	9.78 ± 4.64	0.049*
VAS pain score			
POD1	3.84 ± 1.23	2.60 ± 1.11	<0.001^†^
POD3	3.15 ± 1.15	1.88 ± 0.82	<0.001^†^
POD5	1.55 ± 0.63	1.45 ± 0.57	0.267^†^
POD7	0.95 ± 0.63	1.04 ± 0.66	0.365^†^
Skin temperature around surgical wound (°C)			
POD1	37.26 ± 0.44	37.00 ± 0.50	0.001*
POD2	36.93 ± 0.42	36.78 ± 0.35	0.020*
POD3	36.86 ± 0.38	36.69 ± 0.33	0.002*
POD4	36.78 ± 0.33	36.71 ± 0.40	0.302*
POD5	36.71 ± 0.31	36.65 ± 0.36	0.258*
POD7	36.60 ± 0.30	36.53 ± 0.34	0.233*
Decrease in Hb (g/L)			
POD1	14.10 ± 9.94	18.19 ± 16.37	0.057^†^
POD3	25.67 ± 13.06	24.13 ± 12.72	0.576^†^
POD5	13.22 ± 11.32	13.17 ± 9.88	0.976^†^
HCT at POD3 (%)	29.30 ± 5.14	29.66 ± 4.06	0.626*
Hidden blood loss at POD3 (mL)	233.59 ± 168.93	232.66 ± 142.95	0.970^†^
Postoperative blood transfusion	17	15	0.693^‡^

CSD, closed suction drainage; Hb, hemoglobin; Hct, hematocrit; POD, postoperative day.

* Student *t* test.

^†^ Mann–Whitney *U* test.

^‡^ Pearson Chi‐Square test.

### *Local Inflammation*

Both the VAS pain scores and peri‐wound skin temperature were two quantitative outcomes related to local inflammation. As is shown in Table 3, the average scores of VAS pain at the first postoperative day (POD 1) was 2.60 ± 1.11 and 3.84 ± 1.23 in the non‐CSD and CSD patients; the difference was statistically significant (*P* = 0.000). The average scores of VAS pain at POD 3 was 1.88 ± 0.82 and 3.15 ± 1.15 in the non‐CSD and CSD patients; the difference was statistically significant (*P* = 0.000). Nevertheless, the pain scores of the two groups were found to be nearly identical on POD 5.

The average peri‐wound skin temperature of the patients at POD 1 was 37.00 ± 0.50 and 37.26 ± 0.44 °C in the non‐CSD and CSD groups; the difference was statistically significant (*P* = 0.001). The average peri‐wound skin temperature of the patients at POD 2 was 37.00 ± 0.50 and 37.26 ± 0.44 °C in the non‐CSD and CSD groups; the difference was statistically significant (*P* = 0.020). The average peri‐wound skin temperature of the patients at POD 3 was 36.69 ± 0.33 and 36.86 ± 0.38 °C in the non‐CSD and CSD groups; the difference was statistically significant (*P* = 0.002). There was no statistical significance found between the two groups in respect to peri‐wound skin temperature from POD 4 to POD 7.

### *Blood Loss*

No significant differences were identified in the two groups regarding Hb drop and Hct values at any of the time points (Table 3). Further, there existed no significant difference regarding HBL on POD 3 (Table 3). Postoperative blood transfusion was performed to 17 CSD patients (22.5%, 17/80) and 15 non‐CSD patients (25%, 15/80), which was not statistically significant.

### *Complications*

Postoperative complications were summarized in Table 4. Use of CSD was not associated with a reduction in the incidence of wound haematoma or wound infections. Only a diabetic patient from the non‐CSD group was diagnosed with an incision infection (*Staphylococcus aureus*) on POD 15. Furthermore, no significant difference between the CSD and non‐CSD groups with respect to wound dehiscence, wound erythema, and DVT was observed.

**TABLE 4:** Detailed presentation of postoperative complications

Variables	Patient with CSD (*n* = 80)	Patient without CSD (*n* = 80)	*P* value
All wound infections	0	1	0.316
Wound haematoma	5	9	0.263
Wound dehiscence	2	3	0.650
Erythema of wound	8	7	0.786
Deep vein thrombosis	4	7	0.349

CSD, closed suction drainage.

## Disscussion

Despite the inadequate criteria to recommend its use, CSDs are applied widely in orthopaedics for the intention of preventing the postoperative seroma, and when to remove CSD often influences the discharge time. Nevertheless, patients often report discomfort associated with drains, and drain sites may represent potential sources of infection^18^, ^19^. The efficacy of the prophylactic CSD for orthopaedic surgeries remains inconclusive.

### *Incision Healing and Postoperative Hospital Stays*

Incision healing is associated with period of wound oozing and dressing change. Previous studies involving a meta‐analysis have revealed that CSD facilitates the reduction of wound oozing time and dressing change^20^, ^21^. However, regarding period of wound oozing and dressing change in our results, there was no significant difference between the two groups, which was consistent with the studies on hip arthroplasty^4^, ^22^. A shorter tendency of postoperative hospital stay was seen in ORIF performed without postoperative suction drainage. Patients without CSD favor early activities and are willing undertake quadricep muscle strength exercises. Early mobilization after surgery was conducive to shorter hospital stays, decreased complications, and better functional outcomes^23^.

### *Local Inflammation*

Redness, swelling, heat, and pain are four common clinical signs, which reflect local inflammation^4^, ^24^. Apart from the redness and swelling, the other two signs are easy to measure quantitatively. While the peri‐wound skin temperature was a quantitative result, we measured the four points around the wound at the same time point for the purpose of reducing such relative discrepancies. Our present research indicated that non‐CSD patients were accompanied with the lower peri‐wound skin temperatures from the first to the third postoperative days, revealing that CSD does not suppress peri‐wound skin temperature.

Pain and discomfort during removal of surgical drains is an obvious problem^25^. Before performing this trial, fracture patients felt anxious about the pain from removal of CSD and the rest of the drainage hole. Our results showed that VAS scores were higher in the patients with CSD during the first three postoperative days, indicating that the pain during removal of CSD after ORIF was not alleviated by the perioperative analgesic method. A drainage will hinder daily activities and complicate care work, while the absence of a drainage may facilitate rehabilitation exercises and be conducive to the recovery of patients during the early postoperative period. In a previous report, appropriate pain prevention accelerates rehabilitation and adequate pain relief after CSD increases patient satisfaction, which is associated with shorter hospital stays and more range of femur motion^26^.

### *Blood Loss and Transfusion Requirements*

Provided that drainage placement may increase transfusion requirements, routine drainage use after hip fracture surgery is not recommended^23^. Two studies have indicated a larger proportion of transfusions in patients using CSD, with no related differences of Hb values^27^, ^28^. In our present results, we identified no statistical differences of the study groups regarding Hb drop values, HBL, and postoperative blood transfusion rate in the early postoperative period. These results are in line with those of previous literature^23^, ^27^, ^28^ and can be explained as follows: the administration of intravenous TXA and tourniquet, which inhibits breakdown of clots, may help to reduce blood loss. The hemostasis effect of TXA has been proved in previous research^29^. Thus, all parameters of the blood loss were less than that in previous research and the difference between the two groups may not easily be detected.

### *Complications*

It has been reported that drainage decreases the occurrence of subcutaneous ecchymosis and frequency of dressing reinforcement^6^, ^28^. Nevertheless, the previous results were not in line with our findings. We identified no significant difference of the drainage and non‐drainage groups regarding occurrence of wound erythema, ecchymosis, and dehiscence. The above outcomes reflect leakage of blood and oozing between the fracture ends, which may follow a potential risk of haematoma and infection. Our results reveal that CSD may not decrease these complications. Conversely, some authors have worried that drainage use may increase the infection rate because the infection was significantly related to positive suction tip culture^9^, ^30^.

In our present study, only one patient with diabetes from the non‐CSD group developed the *Staphylococcus aureus* infection, which may have been attributable to the unstable glycaemic control. The reason for less wound infection in our study may involve the following factors. Firstly, our follow‐up period (postoperative 1 month) was inadequate to manifest accurate infection outcomes. Secondly, appropriate selection of prophylactic antibiotics and strictly aseptic techniques may decrease the rate of wound infections. Finally, no significant difference of postoperative DVTs were identified between the two groups, which may be interpreted partly by the daily administration of LMWH started from the day of admission.

### *Strengths and Limitations*

Our present study has two strengths. First, it was designed as a prospective clinical randomized controlled trial and 160 participants were matched according to related preoperative data. Second, few studies have focused on the efficacy of CSD in patients with one‐stage ORIF for acute closed distal femur fractures. However, it is a primary report and all data are measured during hospitalization. Further follow‐up is still needed for evaluating the long‐term prognosis of deep infection and renovation. In addition, some variables that potentially reflect the early recovery were not included, such as the degree of swelling and fracture healing time.

## Conclusions

In this randomized study, non‐use of prophylactic CSD after primary ORIF for closed distal femur fractures was related to less local inflammation and shorter postoperative hospital stay. Prophylactic CSD not only had no significant advantage to minimize blood loss and wound complications, but increased hospitalization costs. Therefore, we suggest that prophylactic CSD after primary ORIF for closed distal femur fractures is not recommended for optimized clinical pathways and accelerated recovery.

### *Availability of Data*

All data are available from the corresponding author upon reasonable request.

## Ethical Approval

The study protocol was approved by the ethics committee of the Third Hospital of Hebei Medical University (NO 2018–026‐1) and is in line with the 1964 Helsinki Declaration and its later amendments or comparable ethical standards.

## Funding

This study was supported by the Innovation Project for Postgraduates of Hebei Province Education Department (grant number: CXZZBS2020123).

## Author Contribution

Yingze Zhang designed the study; Junzhe Zhang and Kuo Zhao searched relevant studies; Junyong Li and Hongyu Meng analyzed and interpreted the data; Yanbin Zhu and Junzhe Zhang wrote the manuscript; and Yingze Zhang approved the final version of the manuscript.
